# Investigating the Link Between Smoking and Fatty Liver via Ultrasound Elastography: A Cross‐Sectional Analysis Study

**DOI:** 10.1155/cjgh/4584914

**Published:** 2026-07-20

**Authors:** Yue Feng, Zhengqing Mu, Changsong Xu, Fan Zhou

**Affiliations:** ^1^ Department of Ultrasound Medicine, The Affiliated Huai’an No. 1 People’s Hospital of Nanjing Medical University, 1 Yellow River West Road Huaiyin District Huai’an, 223300, Jiangsu, China, njmu.edu.cn

**Keywords:** fatty liver, smoking behavior, cotinine, ultrasound, NHANES

## Abstract

**Objective:**

To investigate the link between smoking and fatty liver, particularly the influence of different smoking patterns on its prevalence, aiming to provide insights for public health policies.

**Patients and Methods:**

We conducted a cross‐sectional analysis of data from 6140 adults participating in the NHANES between 2017 and 2020. Participants were categorized into nonsmoking, secondhand smoke exposure, and active smoking groups based on serum cotinine levels. Multivariable regression models were used to assess the associations between smoking status and fatty liver, controlling for potential confounders.

**Results:**

Active smokers showed a significantly higher prevalence of fatty liver compared to nonsmokers, especially among obese subgroups. In obese populations, both active smoking and secondhand smoke exposure remarkably increased the risk of fatty liver (OR = 2.51, *p* = 0.014). In contrast, a negative but statistically nonsignificant association was found between smoking and fatty liver in nonobese individuals.

**Conclusion:**

Therefore, smoking, particularly among obese individuals, is a significant contributor to fatty liver. Raising public awareness of the risks associated with smoking could help enhance liver health and overall quality of life.

## 1. Introduction

Fatty liver arises from an abnormal buildup of lipids within the liver, typically separated into nonalcoholic fatty liver (Pappachan et al. [1]). Over recent decades, its global prevalence has surged, making it the most prevalent liver disorder, especially among obese and diabetic populations (Quek et al. [2]). Data from the American Liver Foundation (2025) indicate that nearly 100 million people in the United States are affected, yet most remain unaware of their status. Untreated, fatty liver can lead to hepatocyte damage and necrosis, progressing to liver fibrosis, which exacerbates disease severity and significantly increases the risk of hepatocellular carcinoma and cirrhosis (Younossi et al. [3]). In addition, the escalating costs tied to hospital care for fatty liver complications generate a considerable economic burden on healthcare systems (Wong et al. [4]). Consequently, it is essential to implement preventive interventions and identify risk factors to curb the impact of this disease.

Tobacco use stands as a leading cause of preventable deaths worldwide (Padron‐Monedero et al. [5]). The World Health Organization (WHO) (2025) reports that over eight million deaths annually are attributed to smoking, including approximately 1.3 million due to secondhand smoke, affecting both smokers and those exposed passively. This underscores the pervasive health risks associated with tobacco exposure, impacting people across all age groups and socioeconomic backgrounds. The link between smoking and the onset as well as progression of fatty liver is intricate, potentially posing a notable health risk (CO [6]; Okamoto et al. [7]). Prior research indicates that smokers are more susceptible to developing fatty liver compared to nonsmokers (Akhavan Rezayat, Dadgar Moghadam, Ghasemi Nour, Shirazinia, Ghodsi, Rouhbakhsh Zahmatkesh, Tavakolizadeh Noghabi, Hoseini, and Akhavan Rezayat [8]; Jung et al. [9]). Harmful substances released during smoking may contribute to liver damage and fat accumulation by promoting oxidative stress and inflammatory responses, thereby facilitating fatty liver development (Colsoul et al. [10]; Donohue [11]; El‐Zayadi [12]; Chen et al. [13]). Cotinine, a biomarker of tobacco use, positively correlates with smoking frequency (Benowitz [14]; Kanamori et al. [15]), and recent studies suggest that elevated cotinine levels may increase the risk for liver fibrosis and fatty (She, Jiang, and Yuan [16]; Kim et al. [17]). This suggests that cotinine not only serves as an important indicator of tobacco exposure but may also be a valuable biomarker for evaluating liver health.

At present, the most commonly used indices to detect fatty liver include the US Fatty Liver Index (USFLI), Fatty Liver Index (HSI), and Fatty Liver Index (FLI) (Ge et al. [18]). However, studies utilizing vibration‐controlled transient elastography (VCTE), which quantifies liver stiffness median (LSM) and controlled attenuation parameter (CAP) for assessing fibrosis and steatosis, are relatively scarce. While USFLI, HSI, and FLI facilitate quantitative evaluations of steatosis risk, their diagnostic accuracy lags behind ultrasound imaging due to the lack of direct imaging data and potential for false‐negative results (Biciusca et al. [19]). In contrast, ultrasound is a safe, noninvasive, and efficient modality that provides more precise measurements of fatty liver and fibrosis through LSM and CAP (Romero‐Gómez and Cortez‐Pinto [20]). Therefore, this study aims to employ ultrasound to elucidate the association between smoking and fatty liver. It seeks to enhance our understanding of fatty liver and its risk determinants and to provide new insights and evidence. Ultimately, this will help refine management strategies for liver disease patients.

## 2. Materials and Methods

### 2.1. Data Sources and Ethical Considerations

The study utilized the NHANES, a comprehensive epidemiological program operated by the Centers for Disease Control and Prevention (CDC), with the primary objective of evaluating health and nutritional conditions across the US population for disease control and prevention [21]. Established in the early 1960s, NHANES employs a sophisticated multistage probability sampling design to ensure nationally representative data collection through periodic surveys for disease control and prevention [22].

The NHANES database encompasses diverse datasets, including participants’ demographic characteristics, biochemical test results, physical examinations, dietary information, and other lifestyle factors. In this study on fatty liver, NHANES data are particularly significant as they provide valuable information for the effective assessment of liver health, including cotinine levels, CAP, and LSM (Unalp‐Arida and Ruhl). By analyzing these data, this research investigates the association between smoking behavior and fatty liver, thereby offering scientific evidence to inform public health policies and clinical practices.

The survey adheres to stringent ethical standards to ensure the rights, privacy, and safety of participants. All participants in NHANES provided informed consent prior to participation, fully understanding the purpose, procedures, and potential risks involved in the survey. This study was exempt from ethical approval.

### 2.2. Study Population

**FIGURE 1:**
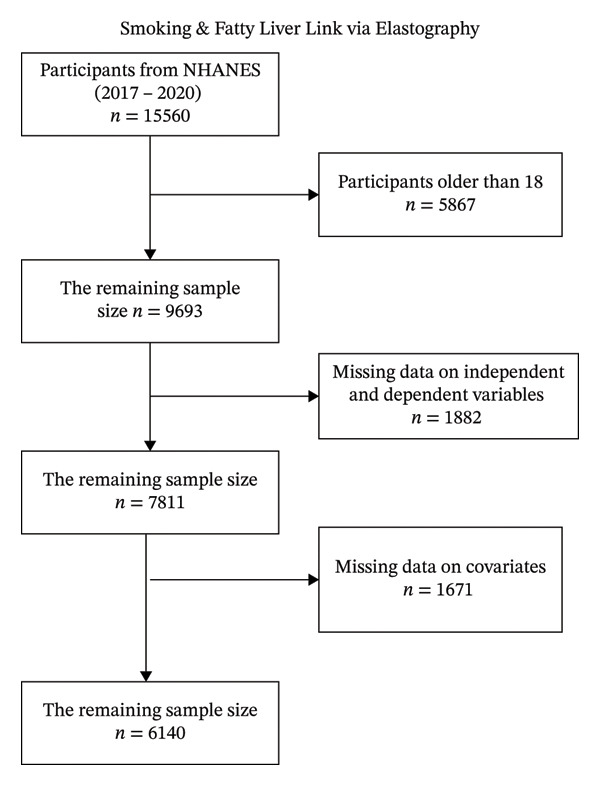
Flowchart of participant inclusion and exclusion based on data from the 2017‐2018 NHANES database.

Data were extracted from the NHANES database (2017–2020). After applying inclusion and exclusion criteria, a total of 6140 participants remained in the final analytic sample. Individuals were eligible if they were at least 18 years old; had full records on CAP and LSM; had complete cotinine data; and had comprehensive information regarding alcohol consumption, body mass index (BMI), and additional covariates (Figure 1).

Cotinine persists longer in the bloodstream than nicotine, making it an ideal biomarker of tobacco use (Lei et al. [23]). The research employed serum cotinine levels as the primary criterion for subject classification: individuals whose cotinine levels were below 0.05 ng/mL composed the nonsmoking exposure group, indicating an absence of both active smoking and secondhand smoke contact. Those with serum cotinine levels ≥ 0.05 ng/mL but < 10 ng/mL formed the secondhand smoke exposure group, reflecting nonsmokers who had encountered secondhand smoke. Finally, participants with cotinine concentrations ≥ 10 ng/mL were identified as the active smoking group, signifying habitual smokers with notably high cotinine levels in the bloodstream (Pirkle et al. [24]; Liu et al. [25]; Tsai [26]; Brody, Faust, and Tsai [27]).

### 2.3. Definitions of Fatty Liver and Liver Fibrosis

Trained health technicians conducted elastography assessments at NHANES mobile examination centers using FibroScan to measure liver stiffness and fat content, evaluating liver health status. During the data processing and editing phase, NHANES staff ensured the integrity and validity of the data, reviewing and correcting any outliers (Health and Survey [28]). These measures contribute to the reliability of the data. Participants were defined as having fatty liver if their CAP was not less than 285 dB/m (Siddiqui et al.[29]; Xiang et al. [30]). Participants were classified as having fibrosis if their LSM was greater than or equal to 7 kPaCenter [31].

### 2.4. Covariates

This study included covariates comprising basic demographic characteristics, laboratory tests, physical examinations, and lifestyle survey questionnaires. Demographic characteristics included gender, age (≥ 18 years), race (with all races categorized as “Other” except for Mexican American, Non‐Hispanic Black, and Non‐Hispanic White), education level, marital status (with “Married” defined as being married or living with a partner), and economic status (assessed using the parent income ratio). Obesity is characterized by a BMI of 30 kg/m2 or higher. During the physical examination, individuals were deemed hypertensive if they had at least one systolic blood pressure reading of 140 mmHg or above, or a diastolic reading of 90 mmHg or higher. Alcohol consumption status was obtained from questionnaires indicating whether participants drank daily, consuming four or five drinks or more. Sedentary behavior was defined as spending over 8 hours a day in seated work, study, or rest. Participants who took part in moderate or vigorous exercise were categorized under “Strong movement.” This included tasks of moderate intensity, causing slight elevations in breathing or heart rate, as well as high‐intensity activities producing notably increased rates, such as continuous lifting or moving heavy objects, digging, or construction work. Diabetes was identified based on self‐reported history in the questionnaire, use of insulin or oral hypoglycemic agents, or fulfillment of clinical criteria such as glycohemoglobin (HbA1c) levels ≥ 6.5% or glucose levels ≥ 126 mg/dL. Stroke status was determined via questionnaires that inquired about a professional medical diagnosis. Coronary heart disease encompassed conditions such as heart failure, myocardial infarction, angina, and coronary artery disease, as confirmed by healthcare provider–validated questionnaires completed by participants.

### 2.5. Statistical Analysis

The NHANES data were collected using a stratified, multistage probability sampling method, and the NHANES sample weights (MEC) were utilized to ensure the national representativeness of the sample for disease control and prevention [32]. When continuous data were skewed, results were summarized by medians and interquartile ranges; for data that followed a normal distribution, means and standard deviations were provided. Participants were categorized into three groups: nonsmokers, those exposed to secondhand smoke, and active smokers. The choice of statistical tests depended on data distribution: one‐way ANOVA was applied to normally distributed variables, and the Kruskal–Wallis H test was used for skewed variables. Chi‐square tests were applied to examine differences in categorical variables.

To explore the association between smoking and fatty liver disease, both unadjusted and adjusted logistic regression models were employed. The unadjusted model excluded covariates, while Model 1 incorporated age, gender, education, and marital status. Model 2 further considered the family income poverty ratio, race, obesity status, alcohol consumption history, sedentary behavior, direct high‐density lipoprotein cholesterol, total cholesterol, and diabetes. We then stratified the analysis by obesity status and applied multivariable regression methods to explore how smoking relates to fatty liver across different subgroups. Smoothing fitting curves were used to visualize and compare changes among these groups. In addition, to consider potential interactions between smoking and covariates and enhance result reliability, stratified logistic regression was conducted for interaction analyses, identifying factors that may influence the smoking–fatty liver relationship in obese populations.

Statistical analyses were performed using *R* software (Version 4.4.1), in conjunction with EmpowerStats (Version 4.2), with statistical significance set at a two‐sided *p* value of < 0.05. To evaluate the strength of each relationship, logistic regression was employed to determine odds ratios (ORs) and their respective 95% confidence intervals.

## 3. Results

### 3.1. Characteristics of the Participants

This study enrolled 6140 participants who were categorized into three cohorts according to serum cotinine levels: nonsmoking exposure group, secondhand smoke exposure group, and active smoke exposure group. As indicated in Table 1, these groups differed significantly in terms of race, education, and smoking status (all *p* < 0.05). The nonsmoking exposure group tended to be older, more educated, and included a higher proportion of married individuals compared to the other two. In addition, this group exhibited not only better economic conditions but also higher rates of alcohol consumption and hypertension. No statistically significant variation was noted in liver fibrosis incidence among the three cohorts (84.53%, 81.71%, 84.47%, *p* = 0.357).

**TABLE 1:** The characteristics of the participants were studied by smoking status.

	**Nonsmoking exposure group**	**Secondhand smoke exposure group**	**Active smoke exposure group**	**p** **value**
Age (years)	50.17 ± 0.73	43.19 ± 1.10	43.12 ± 0.85	< 0.001
Sex (*n*/%)				< 0.001
Male	46.64%	49.50%	59.70%	
Female	53.36%	50.50%	40.30%	
Race (*n*/%)				< 0.001
Mexican American	9.97%	8.36%	4.82%	
Non‐Hispanic White	15.53%	18.10%	14.76%	
Non‐Hispanic Black	67.93%	56.50%	65.75%	
Other	6.57%	17.04%	14.67%	
Education level (*n*/%)				< 0.001
Below high school	7.10%	10.06%	11.98%	
High school or above	92.90%	89.94%	88.02%	
Marital status (*n*/%)				< 0.001
Married	68.76%	50.16%	50.74%	
Unmarried	31.24%	49.84%	49.26%	
Economic status (*n*/%)				< 0.001
Poor	6.56%	14.46%	18.87%	
Not poor	93.44%	85.54%	81.13%	
BMI (kg/m2)	28.7 (25.0, 33.4)	29.7 (25.4, 35.6)	27.3 (23.7, 32.5)	< 0.001
HDL‐C (mg/dL)	53.0 (43.0, 64.0)	49.0 (41.0, 61.0)	50.0 (41.0, 60.0)	< 0.001
TC (mg/dL)	186.0 (161.0, 216.0)	179.0 (156.0, 208.0)	181.0 (155.0, 207.0)	0.015
Sedentary (*n*/%)				0.001
Yes	34.97%	30.74%	27.79%	
No	65.03%	69.26%	72.21%	
Strong movement (*n*/%)				0.222
Yes	79.64%	76.56%	77.56%	
No	20.36%	23.44%	22.44%	
Drinking (*n*/%)				< 0.001
Yes	9.34%	12.44%	25.55%	
No	90.66%	87.56%	74.45%	
HBP (*n*/%)				0.218
Yes	20.78%	22.46%	24.55%	
No	79.22%	77.54%	75.45%	
DM (*n*/%)				0.020
Yes	15.79%	13.46%	12.21%	
No	84.21%	86.54%	87.79%	
CDV (*n*/%)				0.362
Yes	6.22%	5.50%	7.23%	
No	93.78%	94.50%	92.77%	
Fat (*n*/%)				< 0.001
Yes	57.25%	51.10%	63.29%	
No	42.75%	48.90%	36.71%	
Stroke (*n*/%)				0.282
Yes	2.89%	2.48%	3.55%	
No	97.11%	97.52%	96.45%	
LSM (kPa)	5.9 (5.62, 6.09)	6.4 (5.6, 7.2)	5.8 (5.5, 6.1)	0.075
CAP (dB/m)	267.0 (219.0, 308.0)	263.0 (220.0, 317.0)	253.0 (211.0, 301.0)	< 0.001
Fatty liver (*n*/%)				0.023
Yes	61.64%	61.45%	67.32%	
No	38.36%	38.55%	32.68%	
Liver fibrosis (*n*/%)				0.357
Yes	84.47%	81.71%	84.53%	
No	15.53%	18.29%	15.47%	

*Note:* HDL‐C, direct high‐density lipoprotein cholesterol; TC, total cholesterol; DM, diabetes mellitus; CVD, coronary heart disease.

Conversely, the Active Smoke Exposure Group exhibited a notably higher risk of fatty liver (67.32% vs. 61.64% and 67.32% vs. 61.45%, *p* = 0.023). Meanwhile, the proportion of fatty liver cases in the secondhand smoke exposure group did not significantly differ from that of the nonsmoking exposure group.

### 3.2. Association Between Smoking, Fatty Liver, and Liver Fibrosis

Drawing on Table 2, we conducted univariate and multivariate regression analyses using the nonsmoking exposure group as the reference to explore how smoking relates to fatty liver and liver fibrosis. The univariate logistic analysis indicated that individuals exposed to active smoke had a 22% reduced likelihood of developing fatty liver compared to the control group (OR = 0.78, 95% CI: 0.64–0.94, *p* = 0.015). However, upon adjusting for variables in Model 2, this group’s risk of fatty liver increased by 4% relative to the control (OR = 1.04, 95% CI: 0.76–1.44, *p* = 0.796), with the statistical significance diminishing. In addition, though not statistically significant, the active smoke exposure group exhibited a slight elevation in liver fibrosis risk compared to the reference group (OR = 1.13, 95% CI: 0.79–1.61, *p* = 0.520). In Model 1, the secondhand smoke exposure group showed heightened risks of both fatty liver (26% increase, OR = 1.26, 95% CI: 1.03–1.54, *p* = 0.039) and liver fibrosis (35% increase, OR = 1.35, 95% CI: 1.01–1.81, *p* = 0.064).

**TABLE 2:** The association between smoking and fatty liver in the overall population.

	**Coarse model**	**Model 1**	**Model 2**

Fatty liver	OR (95% CI) *p* value	OR (95% CI) *p*value	OR (95% CI) *p* value
Smoking status			
Group 1	Ref.	Ref.	Ref.
Group 2	1.01 (0.81, 1.26) 0.945	1.26 (1.03, 1.54) 0.039	1.14 (0.90, 1.45) 0.304
Group 3	0.78 (0.65, 0.94) 0.015	0.95 (0.78, 1.15) 0.580	1.04 (0.76, 1.44) 0.796

Liver fibrosis	OR (95% CI) *p* value	OR (95% CI) *p* value	OR (95% CI) *p* value
Smoking status			
Group 1	Ref.	Ref.	Ref.
Group 2	1.22 (0.93, 1.59) 0.165	1.35 (1.01, 1.81) 0.064	1.26 (0.93, 1.69) 0.170
Group 3	1.00 (0.78, 1.27) 0.971	1.06 (0.78, 1.44) 0.720	1.13 (0.79, 1.61) 0.520

*Note:* Group 1 refers to the nonsmoking exposure group. Group 2 refers to the secondhand smoke exposure group. Group 3 refers to the active smoke exposure group.

Excess weight or obesity is a key driver in the onset of fatty liver. Table 3 delves deeper into the relationship between smoking and fatty liver among obese individuals. Utilizing a stratified logistic regression model, we found that, within the nonobese subset, both the active smoke exposure group and the secondhand smoke exposure group had a lower risk of fatty liver compared to the control group. Specifically, in the adjusted Model 1, the active smoke exposure group’s risk decreased by 25% (OR = 0.75, 95% CI: 0.58–0.96, *p* = 0.042), and the secondhand smoke exposure group’s risk decreased by 20%, though not statistically significant (OR = 0.80, 95% CI: 0.59–1.09, *p* = 0.179). Conversely, among obese participants, we observed opposing trends. In Model 1, both the active smoke exposure group and the secondhand smoke exposure group exhibited a statistically significant 51% increased risk of developing fatty liver (OR = 1.51 for both, with 95% CIs of 1.06–2.16 and 1.18–1.94, and *p* values of 0.044 and 0.007, respectively). In the adjusted model, the heightened risk persisted as significant for the secondhand smoke exposure group (OR = 1.55, 95% CI: 1.15–2.09, *p* = 0.024).

**TABLE 3:** The association between smoking and fatty liver in the obese population.

	**Group 1**	**Group 2**	**Group 3**

	OR (95% CI) *p* value	OR (95% CI) *p* value	OR (95% CI) *p* value
Nonobese patient			
Coarse model	Ref.	0.68 (0.48.0.98) 0.050	0.61 (0.48.0.76) 0.001
Model 1	Ref.	0.80 (0.59.1.09) 0.179	0.75 (0.58.0.96) 0.042
Model 2	Ref.	0.76 (0.56.1.04) 0.134	0.71 (0.53.0.95) 0.055
Obese patients			
Coarse model	Ref.	1.06 (0.80.1.39) 0.699	1.16 (0.83.1.63) 0.393
Model 1	Ref.	1.51 (1.18.1.94) 0.007	1.51 (1.06, 2.16) 0.044
Model 2	Ref.	1.55 (1.15.2.09) 0.024	1.52 (1.01.2.31) 0.087

*Note:* Through stratified logistic regression analysis, this table uses odds ratios (ORs) along with their 95% confidence intervals (CIs) and *p* values to assess the association between smoking status and fatty liver in obese and nonobese populations.

The differences between the crude and adjusted models suggest that the relationship between smoking exposure and fatty liver was influenced by important confounding factors, particularly obesity, metabolic abnormalities, and lifestyle‐related variables. In the unadjusted analysis, smoking exposure appeared to be associated with a lower risk of fatty liver in the overall population and in nonobese individuals. However, after adjustment for covariates, these associations were attenuated or became nonsignificant, indicating that the crude estimates were likely affected by differences in baseline characteristics across exposure groups. In contrast, among obese participants, smoking exposure remained positively associated with fatty liver, suggesting that obesity may modify the effect of smoking on fatty liver.

### 3.3. Trend Analysis and Subgroup Analysis

We performed a stratified logistic regression and interaction analysis among obese individuals to pinpoint factors that could alter the relationship between smoking and fatty liver. Our results among these participants revealed that diabetes, sedentary behavior, and coronary heart disease were potential modifiers of the smoking–fatty liver association (Table 4). Notably, factors such as sex, age, and race did not significantly impact this relationship. In addition, for those with sedentary lifestyles, active smoking was linked to a striking 151% elevation in fatty liver incidence (OR = 2.51, 95% CI: 1.41–4.46, *p* = 0.014), whereas passive smoking was associated with a 52% increase (OR = 1.52, 95% CI: 1.05–2.19, *p* = 0.056). Smoothing fitted curves (Supporting Materials) further indicated that among nonobese participants, smoking was inversely related to the onset of fatty liver, while in obese individuals—particularly those who were sedentary—smoking showed a positive correlation with fatty liver risk, with a clear pattern of increasing disease prevalence as smoking levels grew.

**TABLE 4:** In obese participants, further stratified logistic regression analysis and interaction effect analysis were conducted.

	**Nonsmoking exposure group**	**Secondhand smoke exposure group**	**Active smoke group**	
Characteristics	OR (95% CI) *p* value	OR (95% CI) *p* value	OR (95% CI) *p* value	P‐interaction
Age				0.972
< 40	Ref.	1.42 (0.80, 2.53) 0.287	1.23 (0.76, 1.99) 0.437	
≥ 40, < 60	Ref.	1.54 (0.91, 2.61) 0.168	1.44 (0.83, 2.49) 0.252	
≥ 60	Ref.	1.24 (0.71, 2.19) 0.481	1.86 (0.63, 5.49) 0.312	
Sex (*n*/%)				0.636
Male	Ref.	1.58 (0.95, 2.63) 0.120	1.34 (0.73, 2.43) 0.374	
Female	Ref.	1.34 (0.96, 1.86) 0.132	1.50 (0.94, 2.40) 0.136	
Race (*n*/%)				0.534
Mexican American	Ref.	1.76 (1.01, 3.07) 0.140	1.32 (0.65, 2.71) 0.500	
Non‐Hispanic White	Ref.	1.10 (0.58, 2.08) 0.793	1.16 (0.56, 2.39) 0.716	
Non‐Hispanic Black	Ref.	1.71 (1.09, 2.69) 0.102	1.49 (0.77, 2.90) 0.325	
Other	Ref.	0.93 (0.62, 1.41) 0.765	1.29 (0.91, 1.81) 0.244	
Education level (*n*/%)				0.934
Below high school	Ref.	1.45 (0.65, 3.22) 0.392	1.47 (0.58, 3.72) 0.441	
High school or above	Ref.	1.44 (1.02, 2.01) 0.074	1.41 (0.89, 2.24) 0.190	
Marital status (*n*/%)				0.510
Married	Ref.	2.13 (1.53, 2.96) 0.003	1.26 (0.81, 1.96) 0.338	
Unmarried	Ref.	0.98 (0.63, 1.54) 0.948	1.52 (0.94, 2.47) 0.134	
Economic status (*n*/%)				0.747
Poor	Ref.	1.40 (0.65, 3.00) 0.415	1.10 (0.52, 2.36) 0.804	
Not poor	Ref.	1.43 (1.05, 1.94) 0.056	1.48 (0.92, 2.38) 0.151	
Drinking (*n*/%)				0.337
Yes	Ref.	1.08 (0.55, 2.13) 0.830	0.86 (0.37, 2.02) 0.741	
No	Ref.	1.49 (1.06, 2.10) 0.056	1.59 (1.04, 2.43) 0.068	
Diabetes (*n*/%)				0.015
Yes	Ref.	1.12 (0.62, 2.04) 0.712	0.63 (0.34, 1.14) 0.172	
No	Ref.	1.51 (1.10, 2.08) 0.040	1.64 (1.04, 2.59) 0.070	
Sedentary (*n*/%)				0.007
Yes	Ref.	1.52 (1.05, 2.19) 0.056	2.51 (1.41, 4.46) 0.014	
No	Ref.	1.30 (0.97, 1.75) 0.114	1.00 (0.65, 1.56) 0.986	
CDV (*n*/%)				0.014
Yes	Ref.	2.87 (1.01, 8.12) 0.088	3.33 (1.87, 5.92) 0.005	
No	Ref.	1.34 (1.00, 1.79) 0.087	1.28 (0.86, 1.90) 0.269	
Strong movement (*n*/%)				0.007
Yes	Ref.	1.45 (1.01, 2.09) 0.086	1.22 (0.82, 1.82) 0.349	0.014
No	Ref.	1.55 (1.00, 2.41) 0.092	0.97 (0.57, 1.64) 0.900	

*Note:* When analyzing all variables, adjustments were made for the following: age, sex, race, education level, marital status, economic status, drinking, sedentary behavior, TC, and HDL‐C.

We performed stratified logistic regression and interaction analyses to determine whether the association between smoking exposure and fatty liver differed according to obesity status and other participant characteristics. The results showed clear heterogeneity between obese and nonobese individuals. Among nonobese participants, active smoking was associated with a lower crude risk of fatty liver, but this association was weakened after covariate adjustment. In contrast, among obese participants, both active smoking and secondhand smoke exposure were associated with a higher risk of fatty liver, and this positive association remained more evident after adjustment, particularly for secondhand smoke exposure. In additional subgroup analyses among obese participants, diabetes, sedentary behavior, and coronary heart disease appeared to modify the relationship between smoking and fatty liver (Table 4). Notably, in participants with sedentary behavior, active smoking was associated with substantially higher odds of fatty liver, and passive smoking showed a similar positive trend. These findings support the presence of interaction effects and suggest that the adverse influence of smoking on liver steatosis may be amplified in metabolically vulnerable individuals, especially those with obesity and physical inactivity.

## 4. Discussion

Globally, fatty liver has become an increasingly important public health problem, especially among individuals with obesity and diabetes. In this study, we examined the association between smoking exposure and fatty liver using NHANES data and ultrasound elastography indicators. Our findings suggest that the relationship between smoking and fatty liver is complex and differs by obesity status.

In the overall study population, the crude analysis showed a higher prevalence of fatty liver among active smokers, but this association was not robust after adjustment for potential confounders. Similarly, in nonobese individuals, some analyses suggested an inverse or weaker association between smoking and fatty liver, which was attenuated after multivariable adjustment. By contrast, among obese individuals, both active smoking and secondhand smoke exposure were associated with a higher risk of fatty liver, indicating that obesity may enhance susceptibility to the harmful hepatic effects of smoking.

Our findings indicate a markedly higher incidence of fatty liver in the active smoking group than in nonsmokers, with this difference reaching statistical significance. This result is consistent with existing literature, suggesting that smoking may lead to liver injury and fat accumulation by promoting oxidative stress and inflammatory responses, thereby increasing the risk of fatty liver (Liu et al. [25]; Caliri, Tommasi and Besaratinia [33]; Jang et al. [34]; Yuan et al. [35]). Notably, the incidence of fatty liver did not differ significantly between nonsmokers and those exposed to secondhand smoke, suggesting minimal impact from secondhand smoke, potentially due to insufficient exposure levels to trigger fatty liver development. In addition, neither active nor passive smoking correlated with liver fibrosis, revealing no intergroup variations. Upon adjusting for various factors, the link between smoking and fatty liver in the general population was insignificant, contrasting with previous studies’ findings. This suggests that while smoking may have some impact on liver health, it may not be a direct cause of fatty liver. The development of fatty liver is primarily attributed to dysregulated hepatic lipid metabolism, which is more directly related to metabolic diseases (Xiang et al. [36]; Zhang et al. [37]).

Additional analyses identified a rising trend in the association between smoking and fatty liver in obese individuals.

Both active smoking and exposure to secondhand smoke notably elevated the risk of fatty liver in this group. These results indicate that in the obese population, smoking may exacerbate the occurrence of fatty liver through other metabolic pathways, particularly in conjunction with a sedentary lifestyle. Smoking generates significant amounts of free radicals and other oxidants, while obesity also increases oxidative stress. Oxidative stress damages the structure of hepatocyte membranes, leading to hepatocyte dysfunction and death while promoting inflammatory responses, thereby further aggravating liver damage and increasing the incidence of fatty liver (Donohue [11]; Karkucinska‐Wieckowska et al. [38]). Sedentary lifestyle is acknowledged as a key contributor to the risk of fatty liver, and our research reveals the interactive effects of smoking and sedentary behavior, indicating that the harm of smoking is significantly heightened in sedentary individuals.

Several biological mechanisms may explain the observed association between smoking and fatty liver, particularly in obese individuals. First, smoking generates large amounts of reactive oxygen species and promotes oxidative stress, which can damage hepatocyte membranes, impair mitochondrial function, and accelerate lipid peroxidation (Burke and FitzGerald [39]; El‐Mahdy et al. [40]). Second, smoking may trigger systemic and hepatic inflammatory responses through the activation of proinflammatory cytokines, thereby contributing to liver injury and steatosis progression (Wu et al. [41]). Third, smoking has been linked to insulin resistance and metabolic dysfunction, both of which are central to the pathogenesis of fatty liver. In addition, smoking may disturb lipid metabolism by increasing lipolysis, altering free fatty acid flux, and promoting hepatic fat accumulation (Artese, Stamford, and Moffatt [42]). These mechanisms may interact with obesity‐related metabolic abnormalities. Obesity itself is associated with chronic low‐grade inflammation, oxidative stress, and insulin resistance. Therefore, when smoking and obesity coexist, their combined effects may further aggravate hepatic lipid deposition and liver injury. This may explain why the association between smoking exposure and fatty liver was more evident in obese participants, especially those with sedentary behavior.

Our study further revealed that, among obese individuals, the link between smoking and fatty liver is shaped by multiple factors, such as diabetes, inactivity, and coronary heart disease. The interplay of these variables introduces complexity to how smoking impacts fatty liver, underscoring the necessity of integrating these possible confounders into public health strategies to precisely determine high‐risk groups and deploy targeted interventions.

This study has several limitations. First, because of its cross‐sectional design, causality cannot be inferred, and the temporal relationship between smoking exposure and fatty liver cannot be established. Future longitudinal studies are needed to confirm these findings. Second, smoking exposure was assessed using serum cotinine measured at a single time point, which may not fully reflect long‐term smoking patterns or cumulative tobacco exposure. Although we adjusted for multiple covariates, residual confounding cannot be excluded. Some potentially important factors, such as dietary habits, detailed alcohol consumption patterns, medication use, and other lifestyle‐related variables, were not fully captured in the present analysis. Third, the study population was derived from NHANES participants in the United States; therefore, the findings may not be fully generalizable to other ethnic, geographic, or clinical populations. Despite these limitations, the use of nationally representative data and elastography‐based liver assessment strengthens the reliability of our analysis.

From a clinical and public health perspective, these findings suggest that smoking exposure may be particularly relevant for fatty liver prevention in obese individuals and those with sedentary lifestyles. Smoking cessation should therefore be considered an important component of risk reduction strategies, together with weight management, increased physical activity, and broader lifestyle modification. These combined interventions may help reduce the burden of fatty liver and its long‐term complications, especially in high‐risk populations.

## 5. Conclusion

In conclusion, the association between smoking exposure and fatty liver appears to vary according to obesity status. In the overall population and in nonobese individuals, the association was weaker, inverse in some crude analyses, or nonsignificant after adjustment. In contrast, among obese participants, both active smoking and secondhand smoke exposure were associated with a higher risk of fatty liver, suggesting that obesity may amplify the adverse hepatic effects of smoking. These findings highlight the importance of smoking cessation and lifestyle intervention, particularly among obese and sedentary individuals, while future longitudinal studies are needed to clarify causality and underlying mechanisms.

## Author Contributions

Yue Feng: conceptualization of this study, methodology, software, validation, resources, and data curation. Zhengqing Mu: formal analysis and writing–original draft preparation. Changsong Xu: writing–review and editing, visualization, supervision, and project administration. Fan Zhou: investigation, writing–review and editing, visualization, and supervision.

## Funding

This research received no external funding.

## Ethics Statement

We conducted our research using data from the National Health and Nutrition Examination Survey (NHANES), adhering to the principles outlined in the Declaration of Helsinki. Approval from the National Center for Health Statistics Institutional Review Board was obtained. As this study involved secondary data analysis, no additional ethical approval was required.

## Consent

Informed consent was obtained from all subjects involved in the study.

## Conflicts of Interest

The authors declare no conflicts of interest.

## Supporting Information

Additional supporting information can be found online in the Supporting Information section.

## Supporting information


**Supporting Information** Supporting Materials. The following supporting information can be downloaded from the website: www.mdpi.com/xxx/s1, Figure S1: Trend analysis of the association between smoking and fatty liver in the obese population and the influence of sedentary behavior.

## Data Availability

The National Health and Nutrition Examination Survey (NHANES) dataset is publicly available from the National Center for Health Statistics at the Centers for Disease Control and Prevention (https://wwwn.cdc.gov/nchs/nhanes/default.aspx, accessed on October 8, 2024).
