# Supplementary material for: Investigating the Link Between Smoking and Fatty Liver via Ultrasound Elastography: A Cross‐Sectional Analysis Study
Source: Can J Gastroenterol Hepatol. 2026 Jul 20;2026:4584914. doi: 10.1155/cjgh/4584914 (PMC13382998; doi:10.1155/cjgh/4584914)

Figure S1. Trend analysis of the association between smoking and fatty liver in the obese population and the influence of sedentary behavior. The vertical axis represents the incidence of fatty liver, while the horizontal axis represents the degree of smoking. In the analysis, adjustments were made for the following variables: Age, Sex, Race, Education level, Marital status, Economic status, Drinking, Sedentary behavior, TC, HDL-C, DM, and Obesity status.


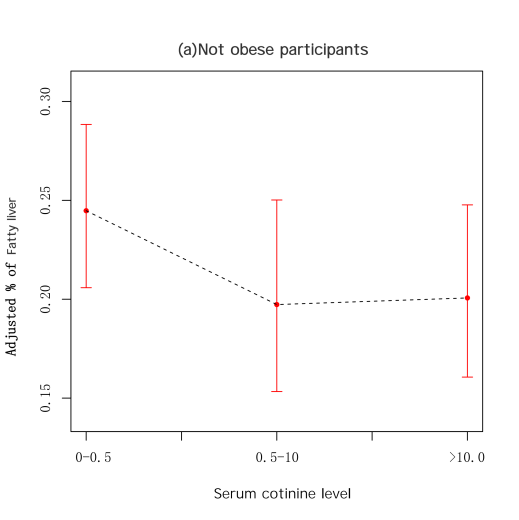

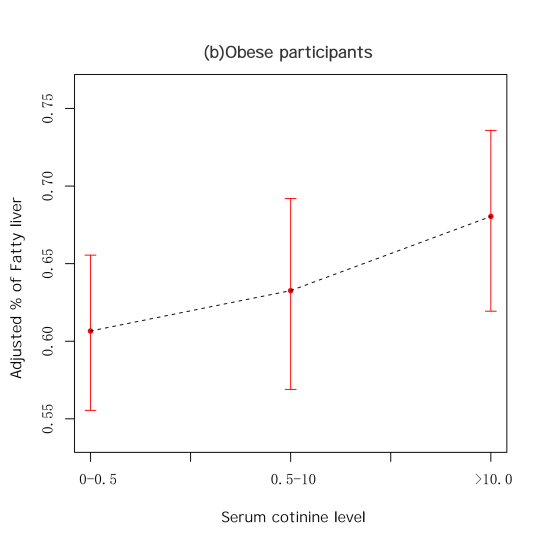

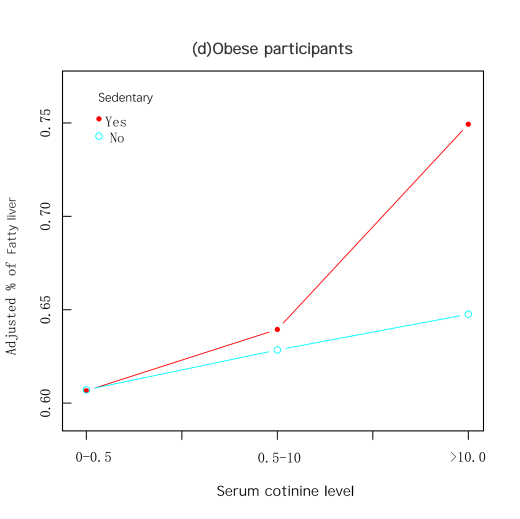

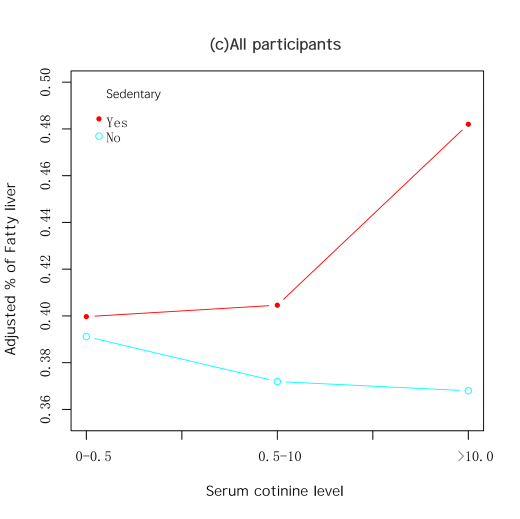

Supplement: Supplementary file 1 — Supporting Information Supporting Materials. The following supporting information can be downloaded from the website: www.mdpi.com/xxx/s1, Figure S1: Trend analysis of the association between smoking and fatty liver in the obese population and the influence of sedentary behavior. [file CJGH-2026-4584914-s001.docx]
